# Classification of position management strategies at the order-book level and their influences on future market-price formation

**DOI:** 10.1371/journal.pone.0220645

**Published:** 2019-08-23

**Authors:** Takumi Sueshige, Didier Sornette, Hideki Takayasu, Misako Takayasu

**Affiliations:** 1 Department of Mathematical and Computing Science, School of Computing, Tokyo Institute of Technology, Nagatsuta-cho, Midori-ku, Yokohama, Japan; 2 ETH Zürich, Department of Management, Technology and Economics, Zürich, Switzerland; 3 Swiss Finance Institute, Geneva, Switzerland; 4 Sony Computer Science Laboratories, Higashi-Gotanda, Shinagawa-ku, Tokyo, Japan; 5 Institute of Innovative Research, Tokyo Institute of Technology, Nagatsuta-cho, Yokohama, Japan; Universidad Veracruzana, MEXICO

## Abstract

Financial prices fluctuate as a results of the market impact of the flow of transactions between traders. Reciprocally, several studies of market microstructure have shown how decisions of individual traders or banks, implemented in their trading strategies, are affected by historical market information. However, little is known about the detailed processes of how such trading strategies at the micro level recursively affect future market information at the macro level. Using a special fined-grained dataset that allows us to track the complete trading behavior of specific banks in a U.S. dollar (USD) versus Japanese yen (JPY) market, we find that position management methods, defined as the number of units of USD bought or sold by banks against JPY, can be classified into two strategies: (1) banks increase their positions by trading in the same direction repeatedly, or (2) banks attempt to reduce their inventories by rapidly shifting their positions toward zero. We then demonstrate that their systematic position management strategies strongly influence future market prices, as demonstrated by our ability using this information to predict market prices about fifteen minutes in advance. Further, by detecting outlier trades, we reveal that traders seem to switch their strategies when they become aware of outlier trades. The evidence obtained here suggests that positions, which are a consequence of historical trading decisions based on the position management strategies of each bank, strongly influence future market prices, and we unravel how market prices at the macro level evolve through an interactive process involving the interaction between well-defined trading strategies at the micro level.

## 1 Introduction

Contrary to the old traditional theoretical assumption that traders are rational and have homogeneous expectations regarding future market prices [1, 2], a number of empirical studies have revealed their heterogeneity at various levels by tracking individual trading behavior [3–11]. The so-called statistical validated network method [7] has provided a classification of traders into several groups and revealed trading synchronization among traders, especially those belonging to government agencies. Lillo et al. [6] showed that the trading activity of the different types of investors (companies, financial, Governmental, Non-profit, Households, and Foreign) is significantly correlated with the flux of news and the daily volatility in a different manner. Sueshige et al. [11] studied individuals’ response patterns to historical market trends and the characteristics of their order submission and transaction frequencies at the level of strategy clusters. These studies mainly demonstrate the heterogeneity inherent in traders’ response patterns to historical market information such as market prices, transaction volumes, and news. However, little is known about the mechanism whereby these strategies at the micro level recursively affect future market prices at the macro level. Within the fruitful picture where financial markets are like an ecology populated by a variety of competing trading strategies [12, 13], it is important to document, not only the variety of trading behaviors but also, the clear roles that these strategies play in determining future market prices.

Using a very fine-grained dataset from a U.S. dollar versus Japanese yen (USD/JPY) market with bank IDs, we document (i) the existence of two types of strategies used to manage positions in terms of the number of units of USD bought or sold against JPY, and (ii) the mechanism by which these position management strategies systematically affect future market prices through an analysis of an individual bank’s trading activity. Focusing on the banks’ response patterns to positional fluctuations, first we show that some banks strengthen their positions by repeatedly trading in the same direction, while other banks attempt to reduce their inventories by alternately trading in opposite directions. The first class of banks are likely to use economically-motivated (EM) strategies, while the second class of banks seems to deploy arbitrage-motivated (AM) strategies. Next, we show that the statistical properties of these two strategies share mathematical characteristics with potential-derived forces (to use an analogy with the concept of forces in Physics): the EM strategy corresponds to a repulsive force, whereas the AM strategy corresponds to an attractive force. Making use of these characteristics, we then empirically demonstrate that the information on such systematic position management allows us to a priori predict future market prices under the condition that banks following either strategy overall have unbalanced positions. Finally, we analyze outlier trades, defined as a series of consecutive small trades on the same side that give cumulatively abnormal large volumes, and show that the counterparties in these outlier trades keep jacking up their limit-order prices in anticipation of future outlier trades.

The rest of this paper is organized as follows. In Section 2, we explain the dataset. In Section 3, we describe the method used to characterize EM and AM strategies, and define outlier trades. In Section 4, we demonstrate the empirical relationship between (i) the unbalanced position and future market price changes, and (ii) the outlier trades and price behavior around these trades. In Sec. 5, we present our conclusions and discuss how positions resulting from historical trading decisions affect future market prices.

## 2 Data description

We first describe the main properties of forex spot markets. Unlike centralized security markets, forex spot markets are decentralized markets, and there are numerous venues to trade currencies such as EBS, Currenex, HotSpot Fxi, FXCM Pro, and FastMatch [14]. The main participants in forex markets are large global banks and financial institutions, and it is said that trading volumes associated with forex trades are the largest of all financial markets. Indeed, the total spot trading volumes reached 5.1 trillion dollars per day in 2016 [15].

In this study, we use high-frequency order submission and transaction data from the USD/JPY market provided by EBS, which is one of the largest trading venues among the forex spot markets. The data cover the week from June 5 to 10 June 2016, during which there were about three million orders submitted. In this dataset, each record contains not only prices, volumes, and timestamps (in milli-seconds), but also anonymized trader and bank IDs. Since these IDs are anonymized but fixed during this period, they allow us to track the complete history of transactions by specific traders or banks over the week. The minimum price unit and the minimum submission volume traders can specify are 0.005 yen and one million dollars, respectively. We denote the price unit as *tpip* which is 0.001 yen (i.e. the minimum price unit traders can specify is 5 tpip), and volumes as multiples of one million dollars. In total, 335 banks undertook transactions totaling about 68 billion dollars during the week.

**Fig 1:**
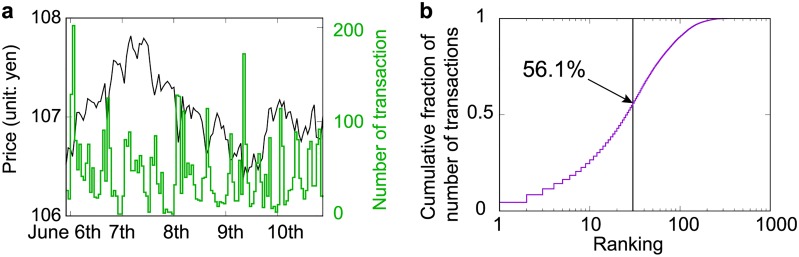
Basic market properties in the week commencing 5 June 2016. (a) Hourly market mid-prices of the USD/JPY pair (black) and number of transactions (green). See S1 Appendix for the distribution of transaction volumes. (b) Fraction of the total number of transactions over the whole week as a function of bank ranking. We order banks by decreasing numbers of transactions. The vertical axis shows the fraction of the total number of transactions by the subset of banks that have ranks equal to or less than the rank given in the horizontal axis. The black line divides the top 30 banks from the other banks, and it can be seen that the transactions of the top 30 banks account for about 56% of total number of transactions. We cumulate positions of traders to whom the same bank ID is attached, and define the total net trader positions as the bank position.

Next, we show the basic statistical properties of our dataset. Fig 1(a) compares the hourly market prices (black) and total number of transactions (green). The left and right vertical axes correspond to market prices and number of transactions, respectively. Fig 1(b) shows that transactions by the top 30 banks accounted for approximately 56% of the total number of transactions. Given that there are 335 banks, it can be seen that less than 10% of the banks accounted for more than 50% of all transactions, and arguably played a dominant role in forex currency trading. See S2 Appendix for the method used to calculate the number of transactions.

Finally, we introduce some terminology in relation to our dataset. Currencies are quoted in pairs such as USD/JPY, where the first (second) currency in a currency pair is called the *base* (*quote*) *currency*. The currency exchange price, for example in a USD/JPY market, represents how much a trader wanting to buy one unit of USD (the base currency) will have to sell in JPY (the quote currency). We refer it as a *long* (*short*) contract to pay (receive) the base currency by receiving (paying) the quote currency. A trader will have several long and short contracts simultaneously. When the accumulated transaction volumes in long (short) contracts are larger than those in short (long) contracts, the trader is deemed to have a net long (short) position. The net long or short position that is constantly exposed to exchange-rate fluctuations is called the trader’s *currency exposure*, or simply exposure. In this study, a long (short) position is denoted by positive (negative) numbers. Trading always requires liquidity providers and liquidity consumers: the liquidity providers act as professional market makers using limit orders, which are unfilled buy and sell orders posted by specifying currency prices, and the liquidity consumers seek prices to buy or sell using market orders, which are buy or sell orders that are executed immediately (see Ref. [11] for details). We refer the former trader as a *maker* and the latter trader as a *taker*. We also introduce *tick* time representing the time counted as a function of the number of transactions.

## 3 Method

### 3.1 Historical average position

**Fig 2:**
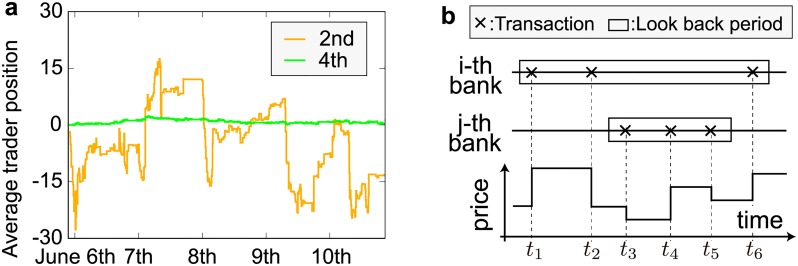
Classification of position management strategies. (a) Average trader positions for the second top (orange) and fourth top (green) banks plotted every 10 minutes. See Fig 1(b) for the definition of the rankings. A positive (negative) number represents a long (short) position in USD/JPY. (b) Schematic of the definition of the historical average position. The historical average position is calculated using the ensemble average of positions observed during past *κ* transactions undertaken by the considered bank (*κ* = 3 in the example). Assume that the *i*-th bank transacted at times *t*_1_, *t*_2_, and *t*_6_, whereas the *j*-th bank transacted at times *t*_3_, *t*_4_, and *t*_5_. The historical average position of the *i*-th bank is the average of the positions at *t*_1_, *t*_2_, and *t*_6_, whereas that of the *j*-th bank is the average of the positions at *t*_3_, *t*_4_, and *t*_5_.

Fig 2(a) shows the position trajectories for two different banks. The vertical axis denotes the average trader position in these banks. A positive (negative) number represents their long (short) positions. The second top bank accumulates a significant position, while the fourth top bank maintains its position very close to 0.

To characterize the position management strategies, we introduce the historical average position. Over a decade ago, Mizuno et al. [16] found that the diffusion speed of market prices was well-characterized by focusing on the price differences between current and historical average prices. Since the trajectories of these two banks appear to show different diffusion speeds, we also introduce the historical average position to characterize their position management strategies in terms of how banks react to position fluctuations from historical levels. Interpretations on the meaning of the historical average position and the gap between a position and the historical average are presented in the conclusion and discussion section. Note that we calculate the aggregate net position using traders sharing the same bank IDs, and regard this as the bank position for the purpose of obtaining an adequate sample size.

The *κ*-tick historical average position is calculated as follows. We define a position during this week as

$$\begin{array}{c} V^{i}\left(t\right)\equiv \sum_{k=1}^{N^{i}\left(t\right)}v^{i}\left(T_{k}^{i}\right) \end{array}$$ (1)

where superscript *i* indicates the *i*-th bank, *N*^*i*^(*t*) is the number of transactions until time *t*, $T_{k}^{i}$ is the *k*-th transaction time during the week, and *v*^*i*^(*t*) is the transaction volume at time *t*. *v*^*i*^(*t*) takes a positive (negative) value when a trader has a long (short) contract. As shown in Fig 2(b), the historical average position over *κ* tick is calculated as

$$\begin{array}{c} {\bar{V}}^{i}\left(t\right)=\frac{1}{\kappa }\sum_{k=0}^{\kappa -1}V^{i}\left(T_{N^{i}\left(t\right)-k}^{i}\right). \end{array}$$ (2)

Since a position has to take an integer value, we also round the historical average position ${\bar{V}}^{i}\left(t\right)$ to an integer, denoted by $\left[{\bar{V}}^{i}\left(t\right)\right]$. In the following analysis, we set *κ* = 15. Note that *κ* values ranging from 10 to 50 provide similar results to those shown in Section 4. However, when *κ* value becomes too large such as 150, we failed to estimate the position management strategies as shown in S3 Appendix.

It is worth mentioning why we focus on analyzing the response pattern instead of the overall positions of all currency pairs. Ideally, the most accurate way to analyze position management strategies is by tracking the positions of all currencies the banks are holding. However, in reality, it is very difficult to track the positions of all currency pairs across all foreign exchange trading venues (e.x. EBS, Currenex, HotSpot Fxi, FXCM Pro, and FastMatch) to which consistent bank IDs are attached. Since the only dataset we are able to access is the USD/JPY market provided by EBS, we analyze the response pattern to position fluctuations, which can be estimated using data from one currency market. See the conclusion and discussion section for a more detailed explanation of why we expect this method to work.

### 3.2 Detection of outlier trades

As depicted by the orange line in Fig 2(a), the position trajectory of the second bank includes several sharp falls and rises, which look like outlier trades to the naked eye. To capture these outlier trades statistically, we use the epsilon (*ϵ*) drawdowns and drawups (EDD) methodology [17–19]. Drawdowns and drawups are traditionally defined as persistent market-price decreases and increases, respectively, over consecutive periods. *ϵ* is introduced to relax the above definition by allowing small market-price deviations from the current drawdowns or drawups up to a predefined tolerance level *ϵ* (see [17–19] for details). Therefore, EDD is able to robustly recognize similar local dynamics, and is useful for detecting local similarities in position trajectories (see S4 Appendix for the detailed reason why we employ EDD to detect outlier trades).

First, we calculate the sum of transaction volumes during [*t*_0_, *t*] (unit: sec):

$$\begin{array}{c} v_{t_{0},t}^{i}\equiv \sum_{\tau \in T_{t_{0},t}^{i}}v^{i}\left(\tau \right), \end{array}$$ (3)

where $T_{t_{0},t}^{i}$ is the set of transaction times for the *i*-th bank during [*t*_0_, *t*]. At each point in time, we need to find the largest position deviation $\delta_{t_{0},t}^{i}$ from the previous extremum.

$$\begin{array}{c} \delta_{t_{0},t}^{i}=\{\begin{array}{cc} \max_{t_{0}\leq l<t}v_{t_{0},t}^{i}-v_{t_{0},t}^{i} & \text{for}\;\text{drawups,}\\ v_{t_{0},t}^{i}-\min_{t_{0}\leq l<t}v_{t_{0},t}^{i} & \text{for}\;\text{drawdowns.} \end{array} \end{array}$$ (4)

This search process ceases at time *t*, when the deviation becomes larger than the predefined tolerance *ϵ*. The time $\tilde{t}=\text{arg}\;{\text{max}}_{t_{0}\leq l<t}\;v_{t_{0},t}^{i}$ $(\tilde{t}=\text{arg}\;{\text{min}}_{t_{0}\leq l<t}\;v_{t_{0},t}^{i}$) determines the end of the drawup (drawdown), and $\tilde{t}+1$ becomes the starting point *t*_0_ for the search for the next drawdown (drawup). The duration of the current drawup or drawdown is defined by $d=\tilde{t}-t_{0}$. Due to this definition, the EDD recognizes that drawups and drawdowns are alternating. We iterate this process until the end of the week. As an initial condition, we start from a drawdown (drawup) period if $v_{t_{S},t_{S}+1}^{i}<0\;\left(>0\right)$ where *t*_*S*_ is the beginning of the week.

Since the typical transaction volume differs from bank to bank, it is reasonable to assume that the tolerance *ϵ* depends on historical fluctuations in the transaction volumes of each bank. Thus we set the tolerance *ϵ* as *ϵ* = *ϵ*_0_ *σ*^*i*^(*t*) where *ϵ*_0_ is a constant and $\sigma^{i}\left(t\right)\equiv \sqrt{\sum_{\tau =t-\omega +1}^{t}{v^{i}\left(\tau \right)}^{2}/\omega }$ is a measure of the volatility of transaction volumes for the *i*-th bank over the time window *w*. As discussed in [20], these partitions are dependent on the introduced parameter (*ϵ*_0_, *ω*). To reduce this dependency, we set *ϵ*_0_ from 0.5 to 5.0 in steps of 0.5 and *ω* from 60 to 300 minutes in steps of 60 minutes (50 samples in all), and define outlier trades in the next step by considering all of the results obtained by each parameter set. See S5 Appendix for detailed data processing before the application of the EDD method.

**Fig 3:**
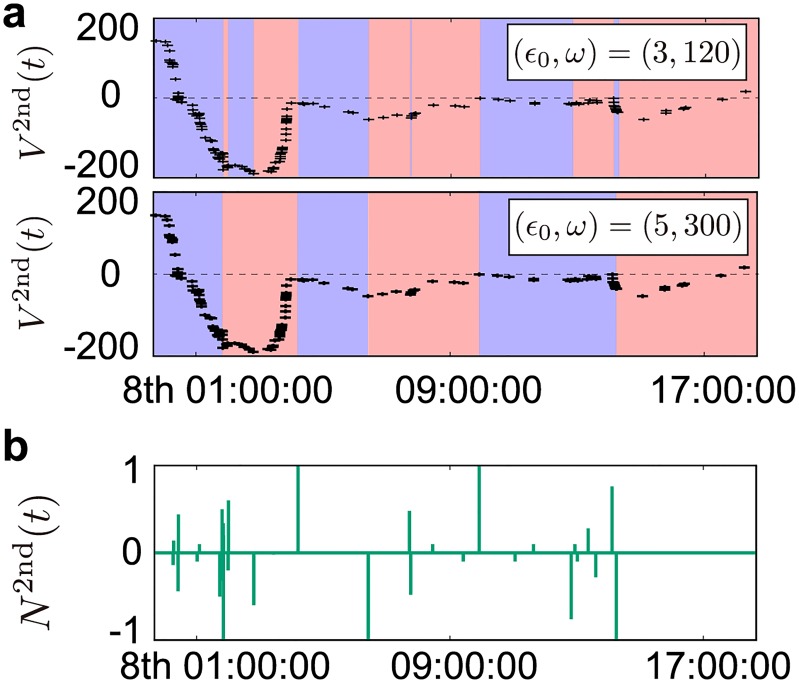
Example of the generation process of the outlier signals *N*^2nd^(*t*) for the second top bank. (a) The position trajectory of the second top bank from 00:00 to 18:00 on 8 June 2016 is shown by the black points. The top (bottom) translucent red and blue boxes represent the drawups and drawdowns, respectively, obtained by the epsilon drawdown (EDD) method (see text) using parameter (*ϵ*_0_, *ω*) = (3,120), and (5,300). (b) Aggregation over a set of parameters (*ϵ*_0_, *ω*) (see text) of the end points of drawups and drawdowns detected by the EDD method for this second top bank, given by Eqs (9), and (11).

Then, we introduce the indicator function to aggregate the end of various drawups and drawdowns. After applying the EDD to the position trajectories, we obtain a set of end dates (unit:sec) and durations (unit:sec) of drawups and drawdowns:

$$ℰU_{\left(\epsilon_{0},\omega \right)}^{i}\;\equiv \;\left\{\tau_{\left(\epsilon_{0},\omega \right)}^{i,{\text{DU}}_{1}},\tau_{\left(\epsilon_{0},\omega \right)}^{i,{\text{DU}}_{2}},\ldots \right\}$$ (5)

$$ℰD_{\left(\epsilon_{0},\omega \right)}^{i}\;\equiv \;\left\{\tau_{\left(\epsilon_{0},\omega \right)}^{i,{\text{DD}}_{1}},\tau_{\left(\epsilon_{0},\omega \right)}^{i,{\text{DD}}_{2}},\ldots \right\}$$ (6)

$$DU_{\left(\epsilon_{0},\omega \right)}^{i}\;\equiv \;\left\{d_{\left(\epsilon_{0},\omega \right)}^{i,{\text{DU}}_{1}},d_{\left(\epsilon_{0},\omega \right)}^{i,{\text{DU}}_{2}},\ldots \right\}$$ (7)

$$DD_{\left(\epsilon_{0},\omega \right)}^{i}\;\equiv \;\left\{d_{\left(\epsilon_{0},\omega \right)}^{i,{\text{DD}}_{1}},d_{\left(\epsilon_{0},\omega \right)}^{i,{\text{DD}}_{2}},\ldots \right\}$$ (8)

where $\tau_{\left(\epsilon_{0},\omega \right)}^{i,{\text{DU}}_{j}\left({\text{DD}}_{j}\right)}$ and $d_{\left(\epsilon_{0},\omega \right)}^{i,{\text{DU}}_{j}\left({\text{DD}}_{j}\right)}$ are the end dates and the duration of the *j*-th drawup (drawdown), respectively, for the *i*-th bank obtained by the parameter (*ϵ*_0_, *ω*). Fig 3(a) shows the example of a series of drawups and drawdowns obtained using parameter sets (*ϵ*_0_, *ω*) = (3, 120), and (5, 300) for the second top bank. The characteristic of the EDD method is that while the EDD with different parameters principally detects different types of local drawups and drawdowns, it occasionally detects the same end points for the global drawups and drawdowns. See S1 Appendix how the EDD changes the size distributions of transaction volumes.

**Fig 4:**
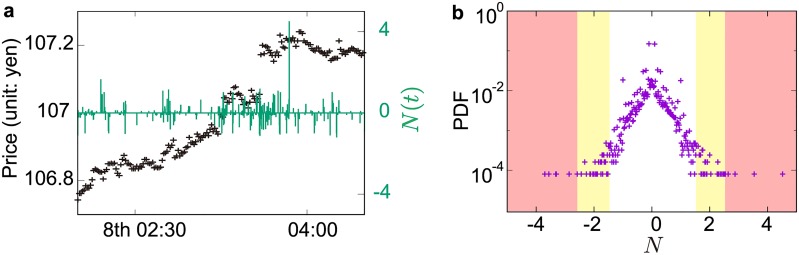
Statistical properties of signals *N*(*t*) recognizing outlier trades, defined by Eq 10. (a) Example of signals *N*(*t*) (green line) on top of the mid-price trajectory (black points). The abscissa represents the time from 2:10 to 4:20 on 8 June 2016. The left and right ordinates represent market prices and the magnitude of signal *N*, respectively. Positive (negative) signals show the end time of outlier trading for the buy (sell) side, and a large *N*(*t*) indicates that several banks concluded their outlier trades simultaneously. (b) Probability distribution function of *N*. We define extreme outliers as being in the bottom and top 0.05% (total 0.1%), highlighted by the translucent red boxes, and define moderate outliers as being in the top and bottom 2.5%, excluding the extreme outliers (total 4.9%), highlighted by the translucent orange boxes.

This information is integrated via the indicator function such that:

$$N^{i}\left(t\right)\;\equiv \;\frac{1}{N_{\epsilon }}\underset{p\in P}{\sum }\left(\underset{\tau \in ℰU_{p}^{i}}{\sum }\delta \left(t-\tau \right)-\underset{\tau \in ℰD_{p}^{i}}{\sum }\delta \left(t-\tau \right)\right),$$ (9)

$$N\left(t\right)\;=\;\underset{i\in ℬ}{\sum }N^{i}\left(t\right),$$ (10)

$$\delta \left(t\right)\;\equiv \;\{\begin{array}{cc} 1 & \text{if}\;t=0,\\ 0 & \text{else}, \end{array}$$ (11)

where *N*_*ϵ*_ is the total number of parameters (in this study, *N*_*ϵ*_ = 50), i.e. the cardinal of P, where P is the set of parameters (*ϵ*_0_, *ω*), and B is the set of analysed banks. The example of this integration process for the second top bank is depicted in Fig 3(b). After the identification of the end dates of drawups and drawdowns obtained by different parameters, we first integrate those end dates over different parameters for a given bank *i* thus obtaining *N*_*i*_(*t*) shown in Fig 3(a), and then integrate them over different banks to create the signal *N*(*t*). We depict a slice of the sample trajectory of *N*(*t*) on top of the mid-prices from 2:10 to 4:20 on 8 June 2016 (Fig 4(a))). The left and right ordinates represent the market prices and the magnitude of signal *N*, respectively. A large signal *N* indicates that several banks concluded their outlier trades at the same time. Seemingly, there is a relationship between mid-price behavior and the magnitude of *N*, especially when *N* exhibits strong signals. We analyze this relationship in Section 4.4.

After summing the indicator function over all parameter sets and banks, we obtain a frequency distribution indicating how many times each *t* is identified as the end of the drawdowns or drawups (Fig 4(b), where *N* = 0 is excluded). The ends of outlier trades are defined as samples exhibiting a strong signal *N*. The extreme outliers are defined as the samples in the bottom and top 0.05% (total 0.1%), while the moderate outliers are defined as the samples in the bottom and top 2.5% excluding the extreme outliers (total 4.9%).

Let us now explain the procedure used to identify the beginning of outlier trades. Let us assume that an outlier trade ends at time *t*. First, we scan ${\mathrm{EU}}_{\epsilon_{0},\omega }^{i}$ $\left(ℰD_{\epsilon_{0},\omega }^{i}\right)$ for a long trade (a short trade) to find the elements equal to *t*. Then, we obtain the corresponding duration from ${\mathrm{DU}}_{\epsilon_{0},\omega }^{i}$ $\left(DD_{\epsilon_{0},\omega }^{i}\right)$ and calculate the beginning by subtracting the duration from the end time: $\tau_{\epsilon_{0},\omega }^{i,{\text{DU(DD)}}_{j}}-d_{\epsilon_{0},\omega }^{i,{\text{DU(DD)}}_{j}}$, which is the beginning of the *j*-th drawup (drawdown) for the *i*-th bank observed for parameter (*ϵ*_0_, *ω*). Note that there are several beginnings associated with the end of one outlier trade because different parameter sets or different banks can provide different beginnings of drawups and drawdowns. In the following analysis, we regard the outlier trade from beginning to end as one long (short) trade when the signal *N* at the end of outlier trades has a positive (negative) value even though it includes several opposite type of small contracts.

## 4 Results

In subsection 4.1, we first classify the position management strategies into two categories: economically-motivated (EM) strategies and arbitrage-motivated (AM) strategies. In Subsection 4.2, we show that the information on the strategy types and the status of the banks’ position allows us to predict ex-ante market prices fifteen minutes ahead. In subsections 4.3 and 4.4, we characterize the position management strategies as well as the outlier trades against market-price behavior, especially those observed around trading decisions. Note that the last two subsections show ex-post statistical properties, and are unrelated to ex-ante market-price predictions.

### 4.1 Classification of position management strategies

**Fig 5:**
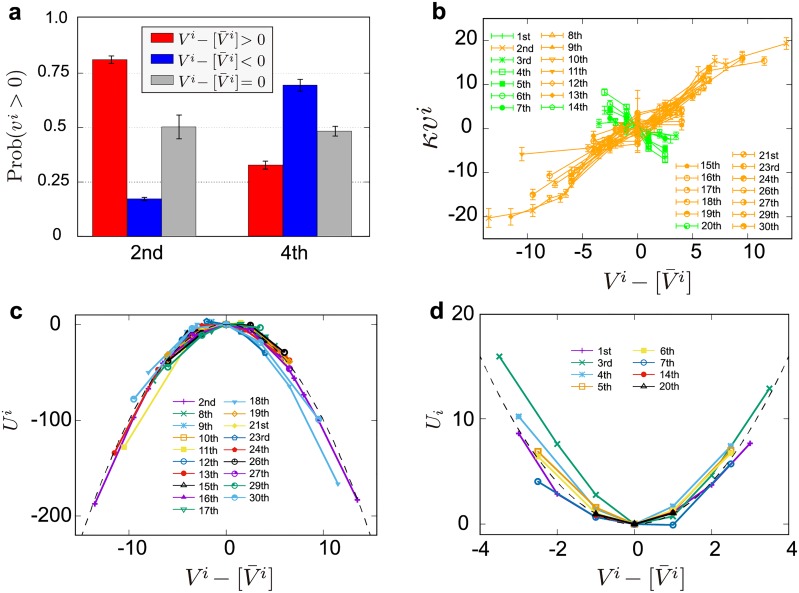
Classification into economically-motivated (EM) strategies and arbitrage-motivated (AM) strategies. (a) Probabilities for the bank to take a long contract in the next time period, conditional on the differences between its current and historical average position (data for the second and fourth top banks). Positive (negative) differences are shown as red (blue) bar plots. When there is no difference, the bar plot is grey. (b) Empirical relationship between the differences in the current and historical average positions and the trading amount *v*^*i*^ of the next transaction. We measure the magnitude of the slopes and the range of fitting errors using gnuplot with the Levenberg–Marquardt algorithm [21]. The orange and green lines show the positive or negative slopes, respectively, after taking fitting errors into account. Note that we aggregated samples to ensure the presence of more than 300 samples in each bin. (c,d) Scaling plots of (b). Repulsive (c) and attractive (d) potential functions collapse onto a quadratic master curve. This figure is obtained for *κ* = 15, which is the number of past transactions over which the averaging over positions is performed (see Eq (2)).

Fig 5(a) shows the probabilities for the second and fourth top banks to have a long contract in their next transaction, conditional on the differences between their current and historical average positions. The red, blue, and grey bars represent positive, negative, and zero differences, respectively, between the current and historical average positions. There is almost an 80% chance that the second top bank will have a long contract in its next transaction when its position leans to the positive, whereas there is less than a 30% chance that the fourth top bank will have a long contract under the same condition. This suggests that position management strategies can be classified into “mean-deviating” and “mean-reverting” strategies.

To verify this statement, Fig 5(b) shows the relationship between the amplitude of the present position unbalance $V^{i}-\left[{\bar{V}}^{i}\right]$ and the next trading volumes *v*^*i*^ for the top 30 banks defined in Fig 1(b). We measure the magnitudes of the slopes and the range of fitting error using gnuplot with Levenberg–Marquardt algorithm [21]. See [22] for more information. The orange and green lines represent the existence of two classes of behaviors, characterised respectively by positive and negative slopes, which are clearly identified as such after taking fitting errors into consideration. Three banks were excluded from this figure because their slopes were not significantly different from 0. This figure clearly demonstrates that the relationship suggested in Fig 5(a) for two banks holds also for the top 27 banks; some banks build their positions, whereas other banks maintain their positions close to 0. The banks characterised by a positive slope build up sequentially their positions and we interpret this as an economically-motivated (EM) strategy. The banks characterised by a negative slope reverse their position, without building inventories. We interpret this behavior as an arbitrage-motivated (AM) strategy. We find that 19 (resp. 8) banks follow the EM (resp. AM) strategy and account for 32.3% (resp. 19.8%) of all transactions during the week.

Based on Fig 5(b), the relationship between *v*^*i*^ and $V^{i}-\left[{\bar{V}}^{i}\right]$ can be described as potential forces in the following functional form:

$$\begin{array}{c} v^{i}\left(t\right)=-\frac{d}{dx}U^{i}{\left(x\right)|}_{x=V^{i}-\left[{\bar{V}}^{i}\right]},\;\;\text{with}\;\;U^{i}\left(x\right)=\frac{b^{i}}{2\kappa }x^{2}, \end{array}$$ (12)

where *b*^*i*^ is constant, and positive or negative *b*^*i*^ corresponds to a mean-reverting or mean-deviating strategy, respectively. To verify this statement, we depicted the potential forces estimated by the above relationship and scaled using *b*_*i*_ and *κ*. Fig 5(c) and 5(d) represent the mean-deviating (c) and mean-reverting (d) strategies characterized by repulsive and attractive potential forces, respectively. To perform a scaling analysis, we used the slopes obtained in Fig 5(b) and shifted the peak of the potentials to the origin in this figure. As can be seen, the potential forces are well-approximated by the quadratic function for the top 27 banks despite the magnitude and signs of the slopes being unique to each bank. This suggests that the position management strategies display similar mathematical characteristics to quadratic potential forces.

We propose to explain the “mean-deviating” strategy (i.e. repulsive potential force) as follows. It is well known that banks split large volumes into small volumes, and subsequently handle these small orders [23]. This strategy further unbalances their positions, which results in the repulsive force. Stop-loss orders are another mechanism that can increase banks’ positions [24]. They are designed to reduce a bank’s potential losses on their positions, and are typically placed in an order book at different prices and volumes and are triggered when market prices reach unfavourable levels relative to their positions. When the stop-loss orders are triggered, the banks will repeatedly trade on the same side to reduce their positions, which also results in a repulsive potential force.

**Fig 6:**
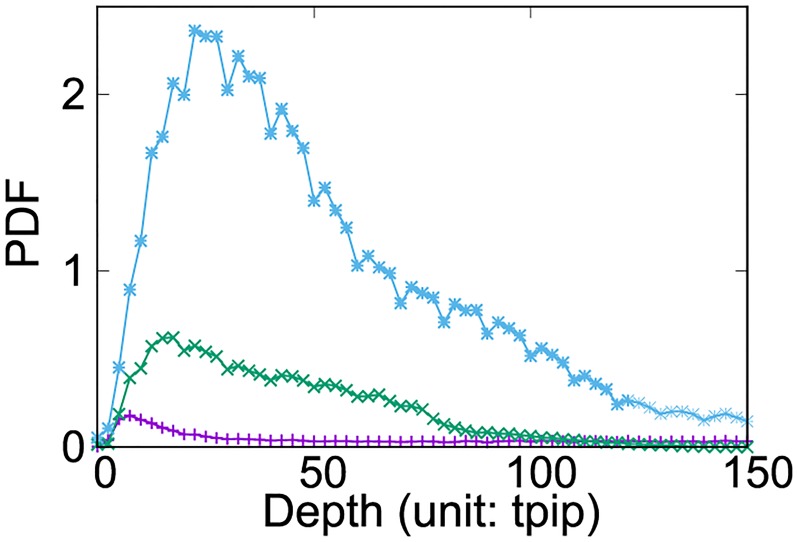
Order-book depth distributions for the banks classified according to their used strategy: Economically-motivated (EM), arbitrage-motivated (AM), and market makers (MM) strategies are represented by violet, green, and light-blue lines, respectively. Depth is the distance from the current best price to the limit-order prices submitted by the banks following each strategy. We use an average for both the bid and ask sides.

Finally, we study the average limit-order book shapes conditional on the banks following the EM and AM strategies, as well as those of the banks not classified into any of the above strategies. Fig 6 shows the average density of bid and ask limit orders residing at specific distances from the best prices (i.e. depth) given in the abscissa. Banks employing the EM and AM strategies are less likely than other banks to submit limit orders over all depths. This implies that the banks not classified into the above strategies mainly work as market makers (MMs) to provide liquidity to markets. Therefore, we refer to their strategy as a MM strategy.

### 4.2 Prediction of future market prices

In this subsection, we demonstrate that the type of deployed position management strategies and the banks’ positions contain information on future market prices, especially when the overall positions held by the banks following EM or AM strategies become unbalanced.

**Fig 7:**
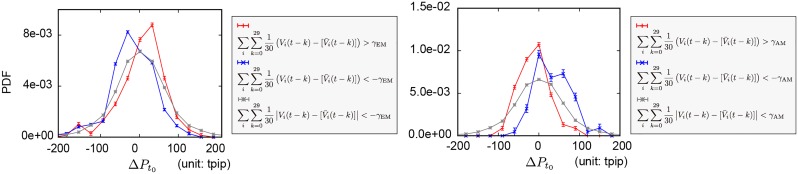
Conditional distributions of market mid-price changes fifteen minutes after the observation of unbalanced positions in banks following either EM or AM strategies aggregated over the past thirty minutes. The EM strategy is depicted in the left panel, and the AM strategy is depicted in the right panel. In the prediction analysis, the basic time unit is a minute. When $\sum_{k=0}^{29}V^{i}\left(t-k\right)-\left[{\bar{V}}^{i}\left(t-k\right)\right]/30$ aggregated over the banks following EM or AM strategies respectively is more (less) than the threshold values *γ*_EM_ or *γ*_AM_ (-*γ*_EM_ or -*γ*_AM_), the distributions are depicted by red (blue) lines. When they are in-between, they are depicted by grey lines. The sampling condition used to detect an unbalanced position is (*γ*_EM_, *γ*_AM_) = (8,6). The average price changes in the distributions depicted by red, blue, and grey lines, respectively, are 4.3 ± 3.6, −18.0 ± 2.8, and 1.3 ± 0.8 tpip for the EM strategy, and −11.5 ± 2.9, 33.0 ± 4.7, and 0.3 ± 0.8 tpip for the AM strategy.

The left (right) panel of Fig 7 shows the distribution of market mid-price changes fifteen minutes after the observation of the unbalanced position $V^{i}\left(t\right)-\left[{\bar{V}}^{i}\left(t\right)\right]$ after aggregating over (i) the banks following the EM (AM) strategy and (ii) the past thirty minutes. The red, blue, grey lines, respectively, correspond to the case where the aggregated position is more than *γ*_EM(AM)_, less than −*γ*_EM(AM)_, and in-between. We can clearly observe skewed distributions, of which the peaks are unique to both the position management strategies and the direction of the position unbalance. The average price changes of the distributions depicted by the red, blue, and grey lines respectively are 4.3 ± 3.6, −18.0 ± 2.8, and 1.3 ± 0.8 tpip for the EM strategy in Fig 7, and −11.5 ± 2.9, 33.0 ± 4.7, and 0.3 ± 0.8 tpip for the AM strategy in Fig 7. The errors represent the standard errors. See S6 Appendix for the robustness of a priori market-price prediction using various parameters (*γ*_EM_, *γ*_AM_) and a different prediction time horizon. The obtained distributions are skewed in the same fashion as shown in Fig 7.

One of the mechanisms generating the skewed distributions is the following. When the aggregated position of the banks following the EM (AM) strategy becomes unbalanced, the banks will simultaneously expand (reduce) their positions by trading in the same (opposite) direction as that of their unbalanced positions. For example, when banks following the EM (AM) strategy overall have long positions, they are likely to simultaneously enter long (short) contracts in their next transaction based on the results in Fig 5(b), 5(c) and 5(d), which drives future market prices upward (downward) and results in skewed distributions, as shown by the red line on the left (right) side of Fig 7. This implies that future market prices are affected by the distribution of the banks’ positions, which is a consequence of historical decision-making processes, and thus such information could be one of the key factors in determining future market prices. See S7 Appendix for an additional test of the future market price prediction using another dataset from 13th to 18th September 2015.

### 4.3 Average price changes around transactions

In Subsections 4.1 and 4.2, we classified the different position management strategies, and showed that the information on the type of deployed position management strategy and on the positions is useful for an ex-ante market price prediction. In this subsection, we ex-post characterize the position management strategies associated with the market-price behavior around the trading decisions (which is unrelated to ex-ante prediction).

**Fig 8:**
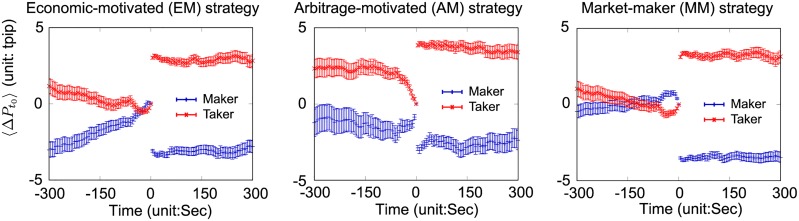
Average price changes around the transaction timing (Time 0) of the EM, AM, and MM strategies as a taker and a maker. Error bars represent the standard errors, and 〈⋅〉 represents the ensemble average. Banks employing the EM strategy are likely to transact as a maker during gradual price appreciation, while banks employing the AM strategy are likely to transact as a taker under rapid price depreciation. No strategy characteristic is evident for the MM strategy. In each case, the average price changes after the transactions plateau, which implies that none of the strategies has strategic superiority on average.

Fig 8 shows the average market prices changes around the transactions as a maker (blue), and as a taker (red) conditional on the bank’s position management strategies. The left, middle, and right panels respectively show the EM, AM, and MM strategies. The price change is defined as

$$\begin{array}{c} \Delta P_{t_{0}}\left(t\right)=\{\begin{array}{cc} P\left(t\right)-P\left(t_{0}\right) & \text{if}\;\text{long}\;\text{contracts,}\\ P\left(t_{0}\right)-P\left(t\right) & \text{if}\;\text{short}\;\text{contracts,} \end{array} \end{array}$$ (13)

where *t*_0_ is the transaction time.

These graphs provide several insights into the position management strategies in terms of the pattern of market-price changes. By considering the slope of the average price changes created by the EM maker strategy, the banks are likely to trade as a maker after a gradual market-price appreciation toward the side the banks are going to hold; they are prone to submitting bid (ask) limit orders when market prices are rising (falling) to follow the current market trends. Given that the EM strategy corresponds to repeated trades on the same side (Fig 5(b)), they seem to focus on reducing a negative market impact on their positions by following market trends and trading as a maker. Since the slope of the average price changes as a taker is much gentler than that as a maker and almost close to zero, it would be difficult to infer taker strategic characteristics associated with market-price behavior.

Looking at the accelerated curve of the average price changes created by the AM taker strategy, we confirm that the banks employing the AM strategy prefer trading as a taker under rapid market-price depreciation toward the side they are going to hold. As Fig 5(b) shows, the AM strategy corresponds to alternate trades on opposite sides, and we can infer the maker strategy as follows. Assume that a bank has a unit of a short contract at a price of 101. When the market price rapidly falls to 100, the bank decides to enter a long contract as a taker to realize immediate gains from the existing short contract. This kind of trading decision process provides a possible explanation for the market-price curve as a taker. The average price changes as a maker’s transactions plateau, and seems to contain almost no information on its strategy.

No peculiar characteristics are evident for the MM strategy in terms of the pattern of market-price changes. Note that the average price changes after the transactions plateau in each case, which implies that, on average, none of the strategies has strategic superiority.

### 4.4 Market price behavior during outlier trades

**Fig 9:**
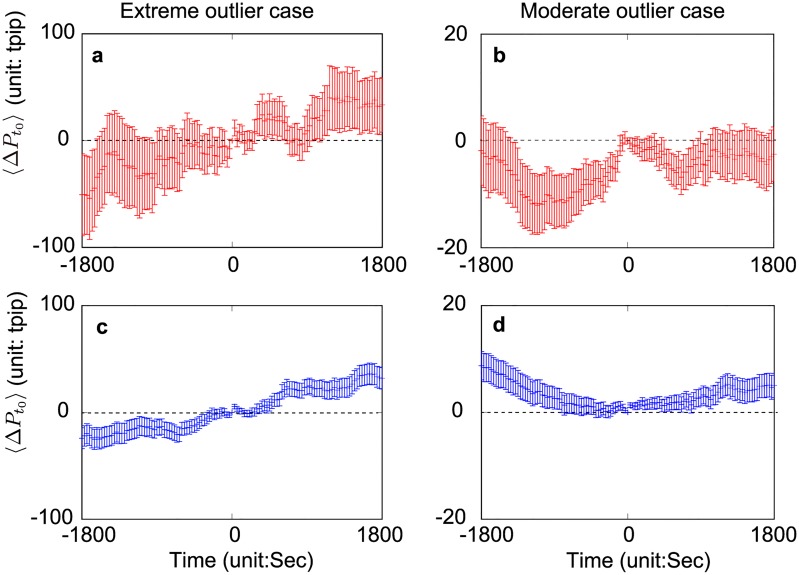
Average price changes around the end (top panels (a,b)) and the beginning (bottom panels (c,d)) of outlier trades. The ordinate represents the average price changes at the end (a,b) and the beginning (c,d) of outlier trades, and the abscissa covers three minutes before and after the end (a,b) and the beginning (c,d) of outlier trades, which corresponds to time 0. We used extreme outliers to make Fig (a,c) with sample size of 12 (a) and 102 (c), and moderate outliers to make Fig (b,d) with sample size of 405 (b) and 1827 (d). Error bars represent the standard errors. Note that the ordinate of the extreme outlier case is five times larger than that of the moderate outlier case. These graphs imply the following two points. First, MMs keep jacking up limit-order prices even after the end of the extreme outlier trades (a), whereas they do not behave in this manner after the end of the moderate outlier trades (b). Second, banks prefer to follow (c) (go against (d)) market-price trends when handling extreme (moderate) outlier trades.

Fig 9 shows the market-price profile around the end (a) and beginning (c) of the extreme outlier trades and the end (b) and beginning (d) of the moderate outlier trades. The prices at *t* = 0 in Fig 9(a) and 9(b) (Fig 9(c) and 9(d)) are the mid-prices observed at the end (beginning) of large volumes. The prices after and before *t* = 0 are the average price transitions from the market mid-prices observed at *t* = 0 (i.e. at the end and beginning of outlier trades). We aggregate both maker and taker trades to ensure there are enough samples to calculate the average price changes.

Fig 9(a) and 9(b) shows the response pattern of the MMs to outlier trades. After the end of large volumes at *t* = 0 in Fig 9(a), the market prices are still increasing without being driven by large volumes. This implies that the MMs recognize the extreme outlier trades, and attempt to jack up limit-order prices in anticipation of the subsequent transactions on the same side. This MM strategy is observed only during extreme outlier trades. As shown in Fig 9(b), the MMs do not behave in the same way during the moderate outlier trades. This implies that the MMs somehow anticipate outlier trades using historical market information and change their strategies to adapt to these outlier events, which is another example of how banks recursively affect future market prices using historical market information. Note that it is an obvious price behavior that market prices increase before *t* = 0 in Fig 9(a) and 9(b) as a result of outlier trades although we have a small change of trend from *t* = −1800 to −1000 in Fig 9(b).

Fig 9(c) and (d) imply that, when traders start to handle extreme (moderate) outlier trades, they prefer to follow (go against) market trends. We conjecture that, when handling extreme outlier trades, banks pay attention to reducing their market impact whereas, when handling moderate outlier trades, they pay more attention to price depreciation than to market impact. As we explained above, it is trivial that market mid-prices increase after *t* = 0 because banks start to handle outlier trades after *t* = 0, which obviously causes market prices to rise. See S8 Appendix for an analysis of how the limit-order book shape changes around the outlier trades.

## 5 Conclusion and discussion

By tracking currency positions representing a detailed history of trading decisions, we classified position management strategies into two classes, namely, economically-motivated (EM) and arbitrage-motivated (AM) strategies, according to the response patterns to position fluctuations. Banks following the EM strategy intensively consolidate their positions, whereas banks following the AM strategy attempt to reduce their positions to close to 0. We further showed that the information on the overall unbalanced positions in either the EM or AM strategies allowed us to predict ex-ante market prices fifteen minutes ahead. In addition, we showed that the banks following a third “market maker” (MM) strategy, defined as the strategy for banks that do not fall in either the AM or EM classes, somehow detect outlier trades using historical market information and change their strategies to jack up limit-order prices in anticipation of the subsequent transaction on the same side.

By this, however, we do not assert that this classification is the only way to characterize trading behaviors. As documented, e.g., in [12], trading strategies have been developed over decades and exhibit a large diversity reflecting different needs. In this context, we have provided a coarse-grained classification, within which many sub-styles and types of strategies can be found.

The evidence presented in this study suggests that banks’ overall positions contain information on future market prices. Studies such as [3–11] have shown that trading strategies are strongly affected by present or past market information such as market prices, volatility, and news. However, we find that banks’ positions, which is a consequence of past trading decisions, also affects future market prices. One possible explanation is as follows. Contrary to the implicit assumption that banks or traders have infinite cash, and thus their default risk can be ignored [1], they are clearly subject to default risk, and thus have to manage their positions. Further, traditional banks have numerous clients, and have to handle large volumes of transactions at the request of their clients, regardless of their expectations regarding future market prices. Such trading behavior, which is required under their business model regardless of their views, will have a strong influence on future market prices, even against their deliberate intention, and thus create an interactive relationship between the evolution of market prices and trading strategies.

**Fig 10:**
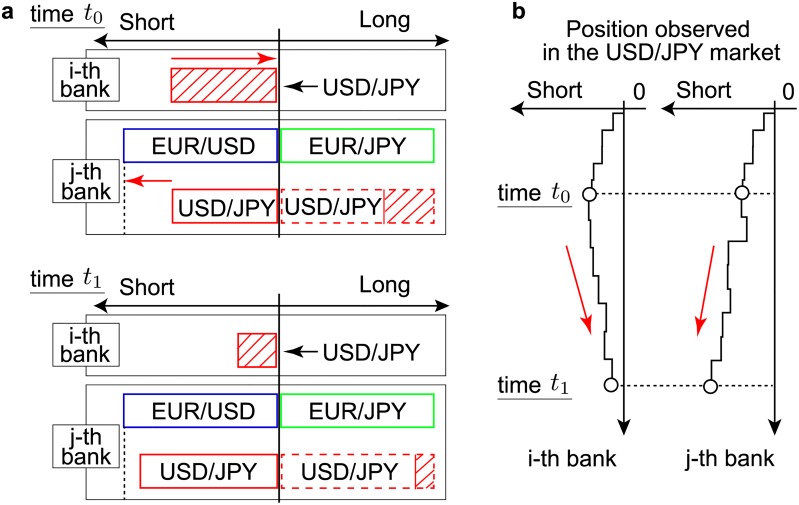
Schematic of the relationship between the positions of different currency pairs that banks manage and their actual exposure. (a) Position profile for the *i*-th bank and the *j*-th bank at time *t*_0_ (top panel) and at time *t*_1_ (bottom panel). The abscissa represents the number of short positions (left arrow) and long positions (right arrow). Assume that the *i*-th bank and the *j*-th bank have the same number of short positions in the USD/JPY market (red) at time *t*_0_. In addition, the *j*-th bank holds a short position in the EUR/USD market (blue), and a long position in the EUR/JPY market (green). The *j*-th bank manages three currency pairs together because the short position in the EUR/USD market and the long position in the EUR/JPY market can be reduced to a long position in the USD/JPY market (see text). We call the USD/JPY position created by using two different currency pairs an indirect position, denoted by a red dashed line in this schematic. Given the direct and indirect positions in the USD/JPY market, the actual exposure (denoted by the red oblique line) of *j*-th bank amounts to the difference in these positions. On the assumption that, on average, both the *i*-th bank and the *j*-th bank attempt to reduce their currency exposure, the *i*-th bank reduces its exposure by long contracts, whereas the *j*-th bank does so by entering into further short contracts, as shown at time *t*_1_. (b) Position trajectories in the USD/JPY market for the *i*-th (left) and *j*-th (right) bank. Despite being in the same position in the USD/JPY market until *t*_0_, the position trajectories differ after *t*_0_ depending on the number of currencies the banks manage together. The trajectory of the *j*-th bank appears to deviate from 0, even though the bank attempts to reduce its currency exposure.

It is worth discussing in more details the meaning of the historical average position. For this, we first explain the possible relationship between currency exposure and the dispersion of position trajectories. As can be seen from Fig 2(a), the second top bank accumulates a significant position, whereas the fourth top bank maintains its position close to 0. One reason for this difference lies in the number of currencies that banks manage simultaneously. Fig 10(a) explains this schematically by using the position profile of some bank (denoted the *i*-th bank) and another bank (denoted the *j*-th bank) at time *t*_0_ (top panel) and at time *t*_1_ (bottom panel). The abscissa represents the number of short position (left arrow) and long position (right arrow). In this schematic representation, we make the following two assumptions:

1. The *i*-th bank and the *j*-th bank have the same number of short positions in the USD/JPY market (red) at time *t*_0_, and the *j*-th bank additionally holds a short position in the EUR/USD market (blue) and a long position in the EUR/JPY market (green)
2. On average, banks are adverse to the risks associated with their positions, in the event of abrupt changes of exchange prices.

When a bank manages its position individually, such as the *i*-th bank, the position represents its level of exposure, and should fluctuate around zero (see the left panel of Fig 10(b)). However, when a bank simultaneously manages its position across several currency pairs, such as the *j*-th bank, the story is different.

The *j*-th bank is able to manage its positions in the USD/JPY, EUR/USD, and EUR/JPY simultaneously because its positions in the EUR/USD market and the EUR/JPY market can be reduced to a position in the USD/JPY market. Consider the example that the *j*-th bank has a short contract in the EUR/USD market at a price of 1 USD, and a long contract in the EUR/JPY market at a price of 130 JPY. In other words, the *j*-th bank pays 1 USD to buy one unit of EUR in the EUR/USD market, and then receives 130 yen to sell one unit of EUR in the EUR/JPY market. These two separate transactions are equivalent to a single transaction involving the purchase of 130 yen to sell 1 USD, which is simply a long contract in the USD/JPY market. We call this position consisting of two different currency pairs an indirect position. See [25–27] for details. Due to this indirect position in the USD/JPY market, the actual exposure the *j*-th bank has to manage corresponds to the difference between the direct and indirect positions, which is presented as a rectangle with oblique lines in Fig 10(a). Recalling the assumption that the banks, on average, behave in a risk-averse manner, the *j*-th bank reduces its exposure by entering into further short contracts, which makes its trajectory deviate from 0 when we look at its position in the USD/JPY market.

Following the previously identified possible relationship between currency exposure and the dispersion of position trajectories, we conjecture that the historical average position approximately corresponds to an indirect position to be offset by a direct position. On the basis of our assumption, banks on average behave in a risk averse manner and control their direct positions associated with indirect positions. Since a direct position would then fluctuate around the level of an indirect position, the recent historical average position becomes a good proxy for its estimation. We applied this method to the top 30 banks shown in Fig 1(b), including not only banks managing several currency pairs simultaneously, but also banks managing a single currency pair. Because the historical average position of the latter banks is supposed to fluctuate around zero, this method is less likely to cause statistical bias in relation to their strategy estimation.

In the above conjecture, we implicitly use timescale differences in the trading frequency between USD/JPY and other currency pairs associated with JPY when classifying position management strategies. Considering the fact that the USD/JPY market provides the highest degree of liquidity compared with all other JPY-related currency pairs, we assume that banks adjust their positions more frequently in the USD/JPY market than in other currency markets. This assumption makes it possible to regard the response pattern to position fluctuations in the USD/JPY market as the overall position management strategy. However, we cannot exclude the possibility that this method would not apply to other currency pairs.

The fact, that we are able to predict market prices about fifteen minutes in advance based on the analysis of how bank strategies influence future market prices, raises the question whether our analysis could be used in the future as the basis for a strategy to arbitrage the market. Indeed, motivated by hefty returns, real traders invest massive effort and resources to construct algorithms to exploit market inefficiencies [28]. If a successful strategy could be developed for a real-time implementation, it is likely that the market impact of this strategy would progressively erase the statistical properties at its basis [13], on a time scale depending on the technical difficulties for implementing the strategy and its level of adoption by market participants. However, we need to emphasise that the information we have unearthed on the relationship between the position management strategies and future price changes has been obtained by using specially tailored datasets that are not available to traders in real-time. Our findings are useful to understand market impact and to provide a robust classification of investment styles at a coarse-grained level. But, it is doubtful that a working strategy can be derived from our analyses, which could be implemented in real-time. However, we cannot completely exclude this possibility as the inventiveness of motivated traders is almost limitless. It would thus be interesting to renew our statistical analysis in the future at different time intervals after the publication of this paper in order to monitor the evolution of the properties that we document here.

In the present article, we introduced the historical average position to estimate existing position management strategies and found that the concept of potentials acting on the position dynamic provided a good description of the data. This motivates the use of the PUCK model [16] to further investigate the dynamics of management strategies in the presence of several currency pairs. In previous work using the PUCK model, the authors also used historical average market prices to detect the potential forces working on the market-price dynamics. Since market prices are created as a consequence of the interactions between traders, it seems natural to assume that there is some type of relationship between the potential forces working at the microscopic scale and those working at the macroscopic scale. We plan to demonstrate the link between these two scales in future works.

In this study, we used trading data with bank IDs, which were specially created for academic use and are not available to real traders. The dataset that real traders are able to access in real time includes information on (i) the latest transaction prices and (ii) the volumes and number of orders up to the 10th price level. It would be interesting to analyze how the results found in this study are related to such open information.

## Supporting information

S1 Appendix Size distribution of large volumes. (DOCX)

S2 Appendix The number of transactions. (DOCX)

S3 Appendix Potential estimation using large *κ*. (DOCX)

S4 Appendix Relationship between the epsilon-drawdown (EDD) method and large volumes. (DOCX)

S5 Appendix Detailed data processing before application of the epsilon-drawdown (EDD) method. (DOCX)

S6 Appendix Future market price distributions. (DOCX)

S7 Appendix Additional testing using dataset from 13th to 18th September 2015. (DOCX)

S8 Appendix Order-book shape after outlier trades. (DOCX)
