# Supplementary material for: Classification of position management strategies at the order-book level and their influences on future market-price formation
Source: PLoS One. 2019 Aug 23;14(8):e0220645. doi: 10.1371/journal.pone.0220645 (PMC6707548; doi:10.1371/journal.pone.0220645)
Supplement: S1 Appendix — (DOCX) [file pone.0220645.s001.docx]

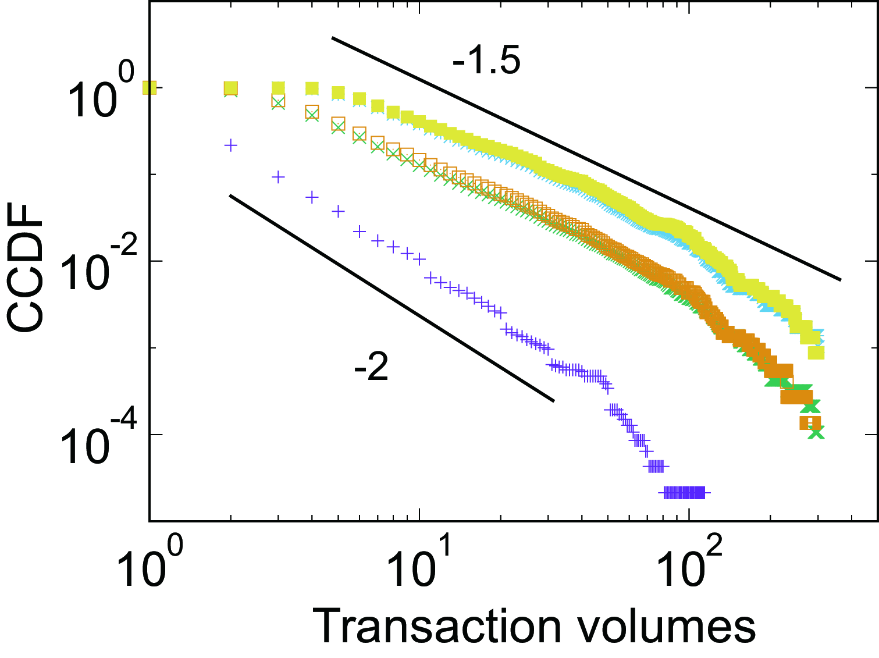
S1 Size distribution of large volumes

Figure 1 Complementary cumulative distribution function (CCDF) of the size of transaction volumes. We order transaction volumes by decreasing values. The vertical axis shows the fraction of transaction volumes with sizes equal to or larger than the one given in the abscissa. The violet pluses denote the CCDF of the original transaction volumes, and the green, light-blue, orange, and yellow symbols correspond to the epsilon-drawdown (EDD) parameter: $\left( \epsilon_{0},\omega\right)=\left( 1,60 \right), \left( 3,60 \right), (1,300)$, and $(3,300)$, respectively.

Fig. 1 shows the complementary cumulative distribution function (CCDF) of the transaction volumes. The violet pluses denote the CCDF of the original transaction volumes. After the application of the epsilon-drawdown (EDD) method with the parameter set $\left( \epsilon_{0},\omega\right)$, we obtain trading volumes different from the original ones. The green, light-blue, orange, and yellow points respectively denote the CCDF of the transaction volumes estimated for $\left( \epsilon_{0},\omega\right)=\left( 1,60 \right), \left( 3,60 \right), (1,300)$, and $(3,300)$. As can be seen, the CCDFs of transaction volumes after the application of the EDD have much fatter tails than that for the original transactions, which is consistent with Ref. [17, 28].
