# Supplementary material for: Classification of position management strategies at the order-book level and their influences on future market-price formation
Source: PLoS One. 2019 Aug 23;14(8):e0220645. doi: 10.1371/journal.pone.0220645 (PMC6707548; doi:10.1371/journal.pone.0220645)
Supplement: S2 Appendix — (DOCX) [file pone.0220645.s002.docx]

S2 The number of transactions

We explain the way to count the number of transactions to define the trading ranking. The complexity lies in the limit-order volumes. When a trader submits one unit, this trader will transact as an either taker or maker. When a trader, however, submits several units, this trader could transact as both a taker and a maker for different part of its submission. We determine that the several submitted units are regarded as two or more different orders and should be counted separately. For examples, the *i*-th bank submits 5 volumes at time 10:00:00, and the bank transacts 3 out of 5 volumes as a taker at time 10:00:00, and 2 out of 5 volumes as a maker at time 10:00:05. We thus consider that the *i*-th bank transacted twice, though the bank submitted only once.
