# Supplementary material for: Classification of position management strategies at the order-book level and their influences on future market-price formation
Source: PLoS One. 2019 Aug 23;14(8):e0220645. doi: 10.1371/journal.pone.0220645 (PMC6707548; doi:10.1371/journal.pone.0220645)
Supplement: S3 Appendix — (DOCX) [file pone.0220645.s003.docx]

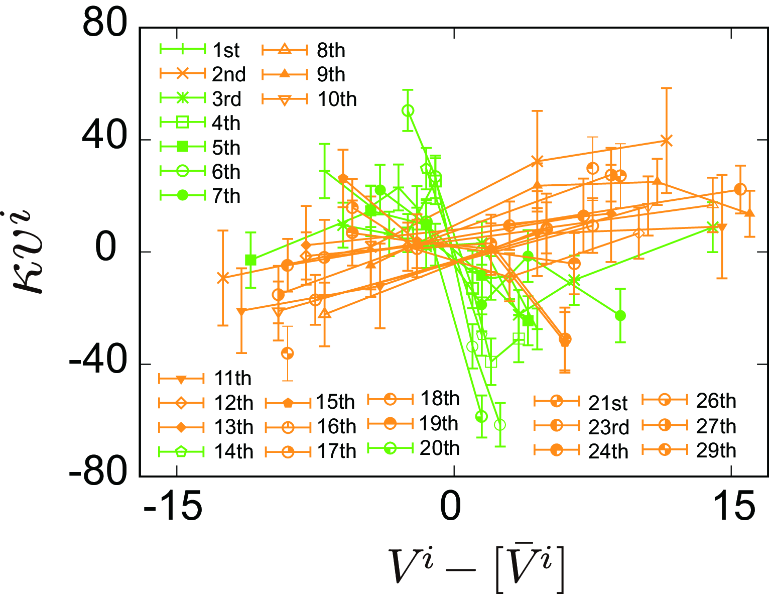
S3 Potential estimation using large $k$

Figure 1 Empirical relationship between the differences in current and historical average position and next trading amount $v^{i}$ for $\kappa=150$, which is the number of past transactions over which the averaging over positions is performed (see equation (2)). The color of the slopes is based on the classification in Fig. 5(b). We aggregated samples to make the number of samples in each bin more than 300. Contrary to the case of $\kappa=15$, we cannot see any meaningful relationship when $\kappa=150$.

Fig. 1 shows the empirical relationship between $v^{i}$ and $V^{i}-[V^{i}]$ by setting $\kappa=150$,

which is the number of past transactions over which the averaging over positions is

performed (see equation (2)). Contrary to the result shown in Fig. 5(b), we cannot see

any specific relationship in this graph, which implies that, to estimate the position

management strategies, $\kappa$ should not be too large.
