# Supplementary material for: Classification of position management strategies at the order-book level and their influences on future market-price formation
Source: PLoS One. 2019 Aug 23;14(8):e0220645. doi: 10.1371/journal.pone.0220645 (PMC6707548; doi:10.1371/journal.pone.0220645)
Supplement: S4 Appendix — (DOCX) [file pone.0220645.s004.docx]

S4 Relationship between the epsilon-drawdown

(EDD) method and large volumes

The EDD method is a useful tool for recognizing a large volume that may be hidden behind a series of small volumes. Since it is well-known that banks fragment large volumes generated by their clients into smaller volumes to minimize the so-called market impact [20], we need to aggregate a series of small volumes to recognize the large volume behind them. By using such a strategy, the local trends in a position trajectory can be regarded as stemming from the small volumes repeatedly posted to the same side. The EDD method is useful for detecting such trends and for compiling segments based on their similarities, corresponding to the period during which large volumes are handled.
