# Supplementary material for: Classification of position management strategies at the order-book level and their influences on future market-price formation
Source: PLoS One. 2019 Aug 23;14(8):e0220645. doi: 10.1371/journal.pone.0220645 (PMC6707548; doi:10.1371/journal.pone.0220645)
Supplement: S5 Appendix — (DOCX) [file pone.0220645.s005.docx]

S5 Detailed data processing before application of

the epsilon-drawdown (EDD) method

We undertake detailed data processing when applying the EDD method to the position trajectories. We apply the EDD to the top 30 banks shown in Fig. 1(b). The shortest timescale is set to one second (sec), and the transactions occurring during one sec are aggregated into the nearest future available times. To reduce the intermittency inherent in trading frequencies [23], we replace non-trading periods within the historical average trading interval. In this analysis, a non-trading period refers to a period of more than ten minutes during which a bank does not trade, and the historical average trading interval is defined as the ensemble average of the transaction intervals observed in the ten minutes prior to the last transaction. We confirmed that the use of other timescales such as 20 or 30 minutes to replace non-trading periods produced similar results and had little impact on the results obtained in Section 3.
