# Supplementary material for: Classification of position management strategies at the order-book level and their influences on future market-price formation
Source: PLoS One. 2019 Aug 23;14(8):e0220645. doi: 10.1371/journal.pone.0220645 (PMC6707548; doi:10.1371/journal.pone.0220645)
Supplement: S6 Appendix — (DOCX) [file pone.0220645.s006.docx]

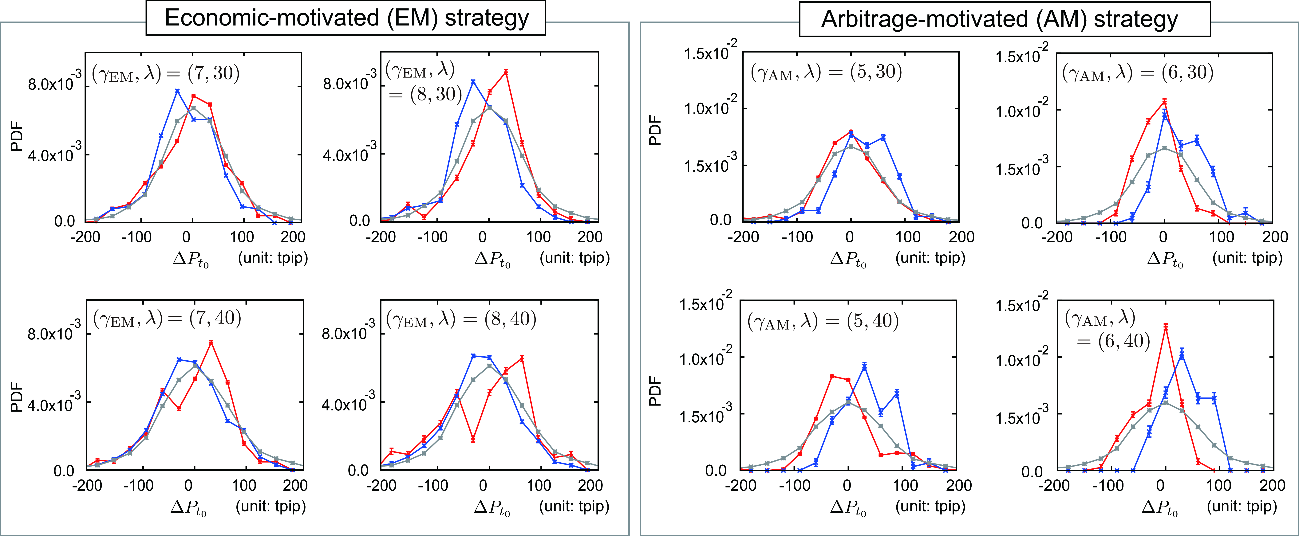
S6 Future market price distributions

Figure 1 Future market price distributions conditional on eight sets of values of the parameters $(\gamma_{\mathrm{EM}\left( \mathrm{AM} \right)},\lambda)$. The left (right)-hand graph is the conditional distributions of market mid-price changes $\lambda$ minutes after the observation of $V^{i}\left( t \right)-[V^{i}(t)]$ aggregated over (i) the past thirty minutes and (ii) the banks following EM (AM) strategy becoming unbalanced against a predefined threshold $\gamma_{EM(AM)}$. When the unbalanced position aggregated over the banks following EM (AM) strategy is greater than the threshold value $\gamma_{EM(AM)}$, or less than $-\gamma_{EM(AM)}$, or in-between, the distributions are depicted by red, blue, and grey lines, respectively. The exact parameter sets regarding $\lambda$, $\gamma_{\mathrm{EM}}$ and $\gamma_{\mathrm{AM}}$ are shown in the top left corner of each panel.

The left (right) panel of Fig. 1 shows the distributions of market mid-price changes $\lambda$ minute after the observation of $V^{i}\left( t \right)-[V^{i}(t)]$ aggregated over (i) the past thirty minutes and (ii) the banks following EM (AM) strategies becoming unbalanced compared with a predefined threshold $\gamma_{EM(AM)}$. The values of $\lambda$, $\gamma_{\mathrm{EM}}$, and $\gamma_{\mathrm{AM}}$ are shown in the top left corner of each panel. The red, blue, and grey lines respectively represent the case where the aggregated unbalanced position is greater than $\gamma_{EM(AM)}$, less than $-\gamma_{EM(AM)}$, or in-between. In each case, the future market prices are skewed in the same fashion such that the red lines in the EM strategy graphs are positively skewed (to the right) and the blue lines in the EM strategy graph are negatively skewed (to the left). The exact opposite behavior can be seen in the AM strategy graphs. See Section 3.2 for details.
