# Supplementary material for: Classification of position management strategies at the order-book level and their influences on future market-price formation
Source: PLoS One. 2019 Aug 23;14(8):e0220645. doi: 10.1371/journal.pone.0220645 (PMC6707548; doi:10.1371/journal.pone.0220645)
Supplement: S7 Appendix — (DOCX) [file pone.0220645.s007.docx]

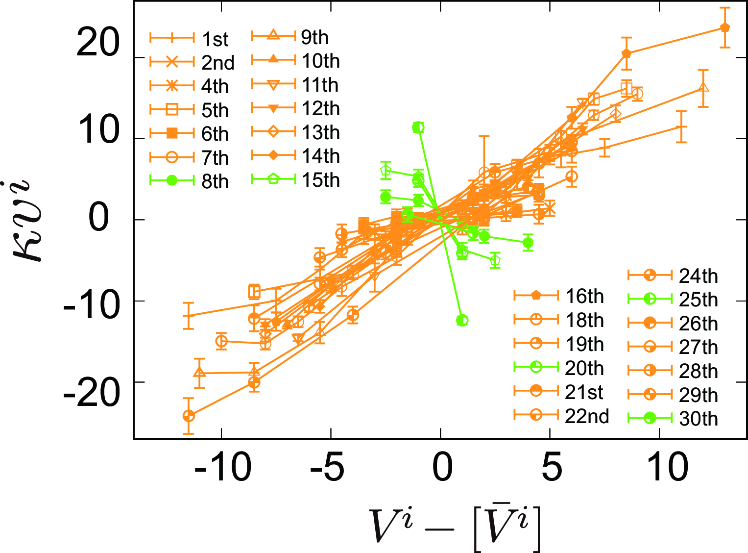
S7 Additional testing using dataset from13^th^ to 18^th^ September 2015

Figure 1 Empirical relationship between the differences in the current and historical average positions and the trading amount $\boldsymbol{v}^{\boldsymbol{i}}$ of the next transaction. The orange and green lines show the positive and negative slopes, respectively. See Fig.5 (b) in the main text for details.

As an additional test, we performed our statistical analysis using the dataset from 13^th^ to 18^th^ September 2015 to check that the following statistical properties also hold as in the main text;
(1) the linear relationship between $V^{i}-[\bar{V}^{i}]$ and $v^{i}$ shown in Fig.5 (b),

(2) the relationship between the position management strategies and future market price changes shown in Fig 7.

As shown in Fig.1 and 2 here, we confirm that the above two relationships are also found for the different week occurring about one year before the dataset used in the main text. This result suggests that the statistical properties (1) and (2) are robust with respect to the choice of the observation period. Note that the parameters defining the lookback period and the thresholds ($\gamma_{\mathrm{EM}}$ and $\gamma_{\mathrm{AM}}$) are different in the two observation periods, but they can be estimated in advance when traders arbitrage financial markets. When dataset covering several consecutive weeks will become available, we will be able to analyze the optimal estimation procedure of these parameters.


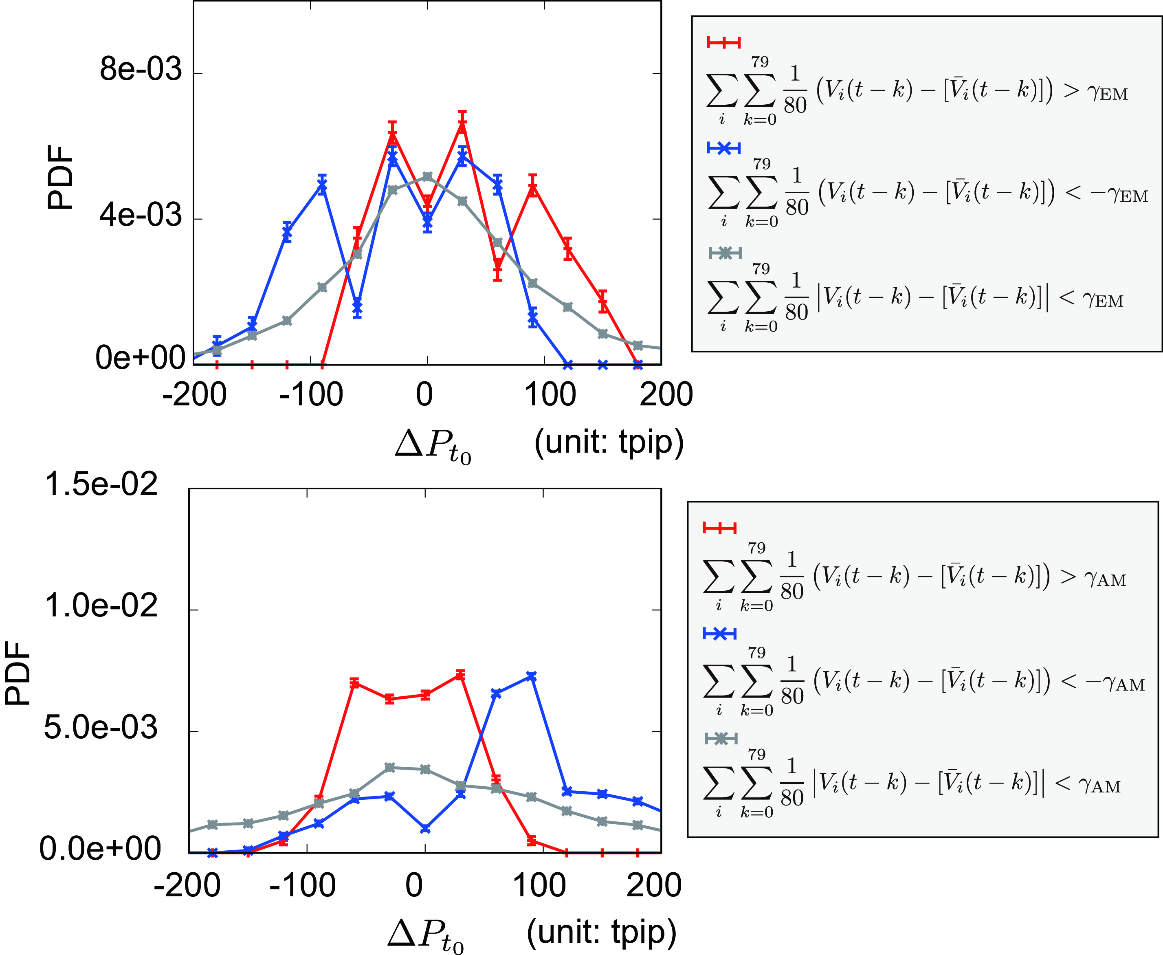


**Figure 2** Conditional distributions of market mid-price changes forty minutes after the observation of unbalanced positions in banks following either EM (top panel) or AM (bottom panel) strategies over the past eighty minutes.

When the unbalanced position is more (less) than the threshold values $\gamma_{\mathrm{EM}}$ or $\gamma_{AM}$ ($-\gamma_{\mathrm{EM}}$ or $-\gamma_{\mathrm{AM}}$), the distributions are depicted by red (blue) lines. When they are in-between, they are depicted by grey lines. The sampling condition used to detect an unbalanced position is $\left( \gamma_{\mathrm{EM}},\gamma_{\mathrm{AM}} \right)=(9,3)$. See Fig.7 in the main text for details.
