# Supplementary material for: Classification of position management strategies at the order-book level and their influences on future market-price formation
Source: PLoS One. 2019 Aug 23;14(8):e0220645. doi: 10.1371/journal.pone.0220645 (PMC6707548; doi:10.1371/journal.pone.0220645)
Supplement: S8 Appendix — (DOCX) [file pone.0220645.s008.docx]

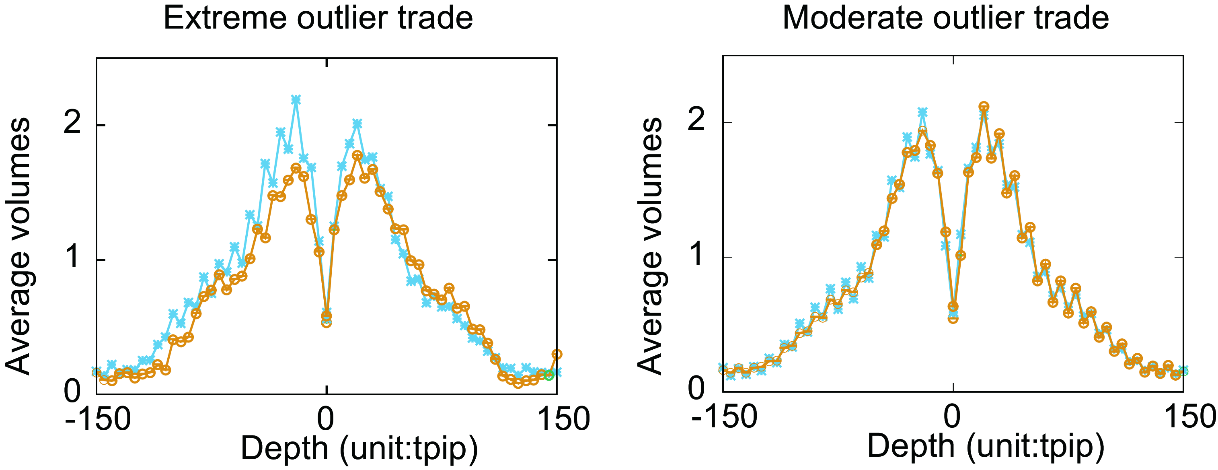
S8 Order-book shape after outlier trades

Figure 1 The left (right) graph shows the average order-book distributions around the end of extreme (moderate) outlier trades. The negative (positive) depth represents the bid (ask) side. The light-blue (orange) line depicts the order-book shape 10 seconds before (after) the outlier trades. The aggregated order-book volumes after the extreme outlier trades are 10% lower than that before the extreme outlier trades, whereas we can see almost no difference in the order book around the moderate outlier trades.

Fig. 1 shows the average order-book distributions of the banks following the MM strategy. The negative (positive) depth means the bid (ask) side. The light-blue and orange lines respectively represent the order-book distribution conditional on the duration of 10 seconds around the outlier trades (i.e. 10 samples for one outlier trade). The height of the distribution after the end of outlier trades is 10% lower than that before the end of outlier trades, which implies that the banks following the MM strategy refrain from posting limit orders when they detect outlier trades. We cannot see order-book differences around the end of moderate outlier trades.
